# Of beta diversity, variance, evenness, and dissimilarity

**DOI:** 10.1002/ece3.2980

**Published:** 2017-05-26

**Authors:** Carlo Ricotta

**Affiliations:** ^1^ Department of Environmental BiologyUniversity of Rome ‘La Sapienza’RomeItaly

**Keywords:** community composition matrix, dissimilarity measures, evenness, species abundances, total sum of squares, variance decomposition

## Abstract

The amount of variation in species composition among sampling units or beta diversity has become a primary tool for connecting the spatial structure of species assemblages to ecological processes. Many different measures of beta diversity have been developed. Among them, the total variance in the community composition matrix has been proposed as a single‐number estimate of beta diversity. In this study, I first show that this measure summarizes the compositional variation among sampling units after nonlinear transformation of species abundances. Therefore, it is not always adequate for estimating beta diversity. Next, I propose an alternative approach for calculating beta diversity in which variance is substituted by a weighted measure of concentration (i.e., an inverse measure of evenness). The relationship between this new measure of beta diversity and so‐called multiple‐site dissimilarity measures is also discussed.

## 1 Introduction

The concept of beta diversity dates back to the work of Whittaker (1960), which coined this term to define the amount of variation in species composition among sampling units (or communities, assemblages, plots, relevés, sites, quadrats, etc.). Since then, the measurement of beta diversity has become a fundamental topic for connecting the spatial structure of species assemblages to ecological processes, such as species coexistence or environmental control (Anderson, Ellingsen, & McArdle, 2006; Tuomisto, 2010a,b).

Given a set of *N* plots, Whittaker (1960) proposed to summarize beta diversity as the ratio of two inventory diversities measured at different scales (i.e., local scale diversity or alpha diversity and regional diversity or gamma diversity), such that β = γ/α, where α is the average diversity of the *N* plots and γ is the total diversity of the pooled set of plots (for details, see Jost, 2007).

An alternative approach, first proposed by McArthur, Recher, and Cody (1966) and recently revitalized by Lande (1996), consists in measuring beta as the excess of regional diversity with respect to local diversity: β = γ − α. However, in both cases, beta diversity is a derived quantity that depends on alpha and gamma (Chao, Chiu, & Hsieh, 2012; Jost, 2007). Therefore, several authors pointed out that it would be desirable to develop a method for calculating beta diversity without reference to alpha and gamma (e.g., Ellison, 2010; Legendre & De Cáceres, 2013).

Among the measures of beta diversity which do not directly depend on alpha and gamma, those based on average dissimilarity between pairs of plots are probably the most commonly used (e.g., Izsák & Price, 2001; Ricotta & Marignani, 2007). However, as emphasized by Diserud and Ødegaard (2007), measures of average dissimilarity across all plots are generally unable to tell us to what extent there is a change in shared species between pairs of plots. To get information on the species shared across more than two plots, so‐called multiple‐site dissimilarity measures (i.e., generalizations of pairwise dissimilarity measures to more than two plots) are required. Examples are the multiple‐site measures of Diserud and Ødegaard (2007), Baselga, Jiménez‐Valverde, and Niccolini (2007), Chao et al. (2012) and Ricotta and Pavoine (2015).

Legendre, Borcard, and Peres‐Neto (2005) and Legendre and De Cáceres (2013) proposed to use the total variance in the community composition matrix of *P* species × *N* plots as a single‐number estimate of beta diversity. This total variance can be calculated either directly or through a dissimilarity matrix obtained using any dissimilarity index suitable for comparing community composition data. However, this method usually calculates variance‐based beta from transformed abundance data. Therefore, it is not always adequate for estimating beta diversity.

In this study, I propose a new approach for calculating beta diversity, inspired by the work of Legendre and De Cáceres (2013) in which variance is substituted by a weighted measure of concentration (i.e., an inverse measure of evenness). The study is organized as follows: First, a short overview on the variance‐based approach is presented. Next, a new index of beta diversity is proposed, which is obtained by averaging the concentration values of single species in the community composition matrix. Finally, to show the behavior of the proposed metric, a worked example is used with data from a belt transect across the beech timberline in the central Apennines (Italy).

## 2 Beta diversity as the variance of community data

Recently, Legendre et al. (2005) and Legendre and De Cáceres (2013) proposed to measure beta diversity as the total variance of a community composition data table. Using a notation similar to that of Legendre and De Cáceres (2013), let **Y** = [*y*_*jn*_] be a community composition matrix containing the presence/absence or the abundance values of *P* species (row vectors **y**_*j*_ = **y**_1_, **y**_2_, … **y**_*P*_ of **Y**) in *N* plots (column vectors **x**_*n*_ = **x**_1_, **x**_2_ … **x**_*N*_ of **Y**). The total variance of the data table, Var(**Y**), can be computed directly from the squared deviations from the row (species) means. Let sjn be the squared difference between the value of species *j* in plot *n*, and the mean value of species *j* such that $s_{jn}={\left(y_{jn}-{\overset{¯}{y}}_{j+}\right)}^{2}$with ${\overset{¯}{y}}_{j+}=\sum_{n=1}^{N}y_{jn}/N$. Summing all values *s*_*jn*_ the total sum of squares of **Y** is obtained:

(1) $$\text{SS}\left(Y\right)=\sum_{j=1}^{P}\text{SS}(y_{j})=\sum_{j=1}^{P}\sum_{n=1}^{N}s_{jn}$$

where $\text{SS}\left(y_{j}\right)=\sum_{n=1}^{N}{\left(y_{jn}-{\overset{¯}{y}}_{j+}\right)}^{2}$. The total sum of squares SS(**Y**) can be directly used to summarize the amount of variation in species composition (or beta diversity) in **Y**. However, transforming SS(**Y**) into the classical unbiased estimator of variance $\text{Var}\left(Y\right)=\text{SS}(Y)/\left(N-1\right)$, a more general measure of beta diversity is obtained, which can be used for comparing data matrices with different numbers of plots (Legendre et al., 2005).

Due to the additive nature of SS(**Y**) and Var(**Y**), both quantities can be partitioned into per‐species contributions (a measure of the degree of variation of individual species across the study area) and per‐plot contributions (a measure of the degree of compositional/ecological uniqueness of single plots).

Given a square *N* × *N* dissimilarity matrix $D=\left[d_{kn}\right]$ of Euclidean distances between plots *k* and *n*, SS(**Y**) can be also obtained as:

(2) $$\text{SS}\left(Y\right)=\frac{1}{N}\sum_{n=1}^{N}\sum_{k>n}^{N}d_{kn}^{2}$$

where *d*_*kn*_ is the classical Euclidean distance $d_{kn}=\sqrt{\sum_{j=1}^{P}{\left(y_{jk}-y_{jn}\right)}^{2}}$. Hence, according to Eq. (2), a different pathway for calculating SS(**Y**) consists in summing the squared Euclidean distances in one half of the dissimilarity matrix **D** and dividing the result by the number of objects N (Legendre & Fortin, 2010; Legendre et al., 2005).

A subtle although relevant shortcoming of this approach recognized by Legendre and De Cáceres (2013) is that the relative dispersion of species abundances within the row vectors **y**_*j*_ that maximizes variance does not coincide with the dispersion of abundances that maximizes beta diversity. An intuitive requirement for beta diversity measures is that beta is maximized if all plots in **Y** do not have any species in common. That is, beta is maximized if all species in **Y** occur only in one plot, meaning that all plots are maximally dissimilar from each other (Ricotta & Pavoine, 2015). Given a hypothetical community composition matrix **Y** composed of four species (*S1–S4*) in four plots (*P1–P4*), for species presence and absence data, if the number of presences for each species is allowed to vary freely and excluding empty species and plot vectors, beta diversity is intuitively maximized at

**Table:** 

	P1	P2	P3	P4
S1	1	0	0	0
S2	0	1	0	0
S3	0	0	1	0
S4	0	0	0	1

whereas SS(**Y**) and Var(**Y**) are both maximized at

**Table:** 

	P1	P2	P3	P4
S1	1	1	0	0
S2	0	1	1	0
S3	0	0	1	1
S4	1	0	0	1

Also, for presence and absence data, the community composition matrix

**Table:** 

	P1	P2	P3	P4
S1	1	1	1	0
S2	0	1	1	1
S3	1	0	1	1
S4	1	1	0	1

produces the same values of SS(**Y**) and Var(**Y**) than the first matrix, whereas, intuitively, the beta diversity of both matrices is substantially different.

Therefore, SS(**Y**) and Var(**Y**) should not be calculated directly on raw species abundances. This is because calculating these quantities on raw species abundances implies that the dissimilarity between pairs of plots is calculated with the Euclidean distance, which is generally considered inappropriate for compositional data. The raw species abundances should be first transformed in ecologically meaningful ways, such as those proposed in Legendre and Gallagher (2001) and Legendre and De Cáceres (2013, Appendix S1). One can then calculate SS(**Y**) from either the transformed species abundance data or from a Euclidean distance matrix **D** calculated from the transformed data.

A consequence of the conceptual difference between variance and beta diversity is that, after data transformation, the relative dispersion of species abundances within row vectors is no longer linearly related to the original dispersion of raw species abundances. To understand why transformed data do not measure the same degree of beta diversity as the non‐transformed data, take, for example, the following matrix with the raw abundances of four species in four plots:

**Table:** 

	P1	P2	P3	P4
S1	10	10	10	0
S2	0	10	10	0
S3	0	0	10	0
S4	0	0	0	10

After Hellinger transformation (i.e., one of the “appropriate” data transformations listed in Legendre & De Cáceres, 2013, Appendix S1), which consists in transforming the raw abundances *y*_*jn*_ into relative values per plot by dividing each value by the plot sum $y_{+n}=\sum_{j=1}^{P}y_{jn}$ and then taking the square root of the resulting values such that $y_{jn}^{\prime }=\sqrt{y_{jn}/y_{+n}}$, we obtain the transformed matrix:

**Table:** 

	P1	P2	P3	P4
S1	1	0.71	0.58	0
S2	0	0.71	0.58	0
S3	0	0	0.58	0
S4	0	0	0	1

in which the transformed species abundances within rows are no longer linearly related to the original ones.

This nonlinear relationship between the raw and the transformed species abundances may be a problem for a correct partition of beta diversity into per‐species and per‐plot contributions. For instance, the preservation of the linear relationship between the relative dispersion of species abundances within row vectors after data transformation is a crucial aspect of the calculation of beta diversity. As shown by Eq. (1), beta is obtained as the sum of the squared deviations from the means of single species regardless of the abundances of the other species, meaning that the species vectors **y**_*j*_ of **Y** act as independent units for the calculation of beta diversity (see also Ricotta & Pavoine, 2015).

A different solution consists in calculating SS(**Y**) with Eq. (2) using dissimilarity indices other than the Euclidean distance. These indices, which were developed to summarize plot‐to‐plot dissimilarity from many different perspectives and motivations, should conform to a set of properties listed in Legendre and De Cáceres (2013) that render them adequate for summarizing beta diversity. Like in the previous case, this operation implies some sort of nonlinear standardization of the raw abundance data in **Y** by row sums, column sums, or both, which necessarily change the relative dispersion of species abundances within row and column vectors (Anderson et al., 2006). This transformation is performed automatically by the index. Therefore, computing the total sum of squares SS(**Y**) from a dissimilarity matrix **D** using an appropriate dissimilarity coefficient other than the Euclidean distance equals to transforming the original community composition matrix **Y** to a new matrix **Y′** = [*y*′_*jn*_] and then computing SS(**Y**′) from the new species abundances $y_{jn}^{\prime }$ (Legendre & Fortin, 2010). From SS(**Y**), one can then compute Var(**Y**) in the usual way by dividing SS(**Y**) by (*N* − 1).

Note that calculating beta diversity with Eq. (2) is conceptually identical to the usual way of obtaining beta diversity from the average dissimilarity between pairs of plots. The only difference is that the average dissimilarity between pairs of plots ${\overset{¯}{d}}_{kn}$ is usually calculated by summing all pairwise dissimilarities (not necessarily Euclidean distances) *d*_*kn*_ between plots *k* and *n* in **D** (with *k* ≠ *n*) and then dividing the result by $N\times \left(N-1\right)\;:\;{\overset{¯}{d}}_{kn}=1/N\left(N-1\right)\sum_{n=1}^{N}\sum_{k=1}^{N}d_{kn}$. By contrast, in Eq. (2), only the upper or lower half of the dissimilarity matrix is considered, such that $\text{Var}\left(Y\right)=1/N\left(N-1\right)\sum_{n=1}^{N}\sum_{k>n}^{N}d_{kn}$. Accordingly, both quantities differ only by a factor two: ${\overset{¯}{d}}_{kn}=2\text{Var}\left(Y\right)$.

The key lessons learned from this short overview are that: (1) The total variance of the raw community composition matrix does not provide a correct estimate of beta diversity because the relative dispersion of species abundances that maximizes beta diversity does not correspond to the dispersion that maximizes variance. Chao and Chiu (2016) showed that, although the calculation of the total variance of the raw community composition matrix does not necessarily require α and γ formulas, nonetheless variance is implicitly constrained by α, γ, and the total species abundances in **Y**. Therefore, it cannot be compared across multiple sets of communities with different α, γ, or total species abundances. Before beta diversity is computed, the raw species abundance data in **Y** should be transformed in an appropriate, usually nonlinear way. This transformation will thus affect the partition of beta diversity into per‐species and per‐plot contributions.

(2) The average dissimilarity between pairs of plots ${\overset{¯}{d}}_{kn}$ represents an adequate way for calculating beta diversity directly from raw species abundances, provided that the selected dissimilarity coefficients conform to a set of empirical properties listed in Legendre and De Cáceres (2013). Half this quantity can be also interpreted as the variance of a new (usually unknown) matrix **Y′** = [*y′*_*jn*_] obtained by nonlinear transformation of the original community composition matrix **Y**. However, being based on plot‐to‐plot dissimilarities, this “distance‐based option” does not allow to decompose overall beta diversity into the contributions of individual species or plots.

In the following sections, building on Legendre and De Cáceres (2013), I will show that beta diversity can be adequately summarized by a weighted average of the concentration values of the species vectors **y**_*j*_ of **Y**. The proposed method gives rise to a new family of multiple‐site dissimilarity measures, which preserve the relative dispersion of species abundances within rows.

## 3 Beta diversity as the weighted concentration of community data

Given a community composition matrix $Y=[y_{jn}]$ containing the presence/absence scores, number of individuals, cover or biomass values of *P* species in *N* plots, to coherently frame the notion of beta diversity, I will start from three fundamental requirements that an index β in the range 0–1 should meet to reasonably behave in ecological research: (1) β takes the value one, denoting maximum diversity, if all species in **Y** occur only in one plot; (2) β takes the value zero, denoting minimum diversity if each species occur in all plots with the same abundance; (3) the species vectors **y**_*j*_ of **Y** should act as independent units for the calculation of beta diversity, meaning that each species should contribute to beta diversity regardless of the abundance of the other species in **Y**. The first two requirements are related to the extreme values of β, while the third requirement makes a distinction between classical measures of beta diversity and measures of ecological complexity, which take into account the amount of “correlation” between the system components, such as the degree of co‐occurrence between species and their spatial arrangement (for details, see Ricotta & Anand, 2006).

Hence, for calculating the overall beta diversity of the community composition matrix **Y**, we first have to calculate the beta diversity of single row vectors β(**y**_j_). To this purpose, we need a family of measures attaining their maximum values if species *j* occurs only in one plot and its minimum value if *j* occurs in all *N* plots with equal abundance. This is usually performed with concentration measures. These measures, also known as dominance or inequality measures, are typically expressed as the complement of evenness, with indices of evenness being basically relative diversity measures or normalizations of diversity measures in the range 0–1. Given a set of Q objects with relative abundances *p*_*i*_ (*i *=* *1, 2, …, *Q*) such that 0 ___ *p*_*i*_ ___ 1 and $\sum_{i=1}^{Q}p_{i}=1$, evenness measures quantify the equality of the relative abundances of the Q objects, maximum evenness arising for an equiprobable object distribution, and the more the relative abundances of objects differ the lower the evenness is. While in ecology, evenness is traditionally used for calculating the equality of *P* species in one single plot, here I suggest to use the complement of evenness to quantify the (in)equality of the relative abundances of one single species in the *N* plots.

The ecological literature is full of evenness measures with different properties and different sensitivity to rare and common species (Hill, 1973; Jost, 2010; Ricotta, 2003), such that the practitioner can select the index that best matches his specific requirements. Among the multitude of available evenness measures, Pielou's (1966) index seems an adequate choice for estimating beta. First, the raw abundances *y*_*jn*_ in each row are normalized into relative values by dividing each value by the row sum $y_{j+}=\sum_{n=1}^{N}y_{jn}$ such that $p_{jn}=y_{jn}/y_{j+}$ This data transformation preserves the relative dispersion of abundances within species vectors. Next, Pielou's evenness of each row is calculated as *EVE*(**y**_*j*_) = *H*(**y**_*j*_)/log *N*, where $H\left(y_{j}\right)=-\sum_{n=1}^{N}p_{jn}\log p_{jn}$ is the Shannon entropy of species **y**_j_ and *N* is the number of plots in the community composition matrix. The beta diversity of single‐species vectors is then obtained as:

(3) $$\beta \left(y_{j}\right)=1-EVE\left(y_{j}\right)$$

For a fixed number of plots *N*, β takes the value one if species *j* is present only in one plot with relative abundance *p*_*jn*_ = 1 and the value zero if *j* is present in all plots with relative abundance 1/*N*. Note that β(**y**_j_) can be interpreted as a rescaled version of Theil's (1967) inequality measure Th(**y**_*j*_) = log *N* − *H*(**y**_*j*_) used in econometrics for summarizing the inequality of household incomes. For instance, according to Eq. (3) β(**y**_*j*_) = 1 − *H*(**y**_*j*_)/ log *N* = Th(**y**_*j*_)/ log *N*.

Finally, the total beta diversity of *Y* can be obtained as the weighted average of the single‐species values β(**y**_j_):

(4) $$\beta \left(Y\right)=\sum_{j=1}^{P}w_{j}\times \beta \left(y_{j}\right)=1-\sum_{j=1}^{P}w_{j}\times EVE\left(y_{j}\right)$$

the weights *w*_*j*_ (with 0 ≤ *w*_*j*_ ≤ 1 and $\sum_{j=1}^{P}w_{j}=1$) can be determined according to the users’ requirements within the specific context of the analyses. If all species are considered equally important, like for presence and absence data, the weights can be uniformly set to 1/*P*. On the other hand, for species abundance data, a reasonable approach is to set the weights proportional to the total species abundances within the community composition table, such that *w*_*j*_ = *y*_*j*+_/*y*_++_ where $y_{++}=\sum_{j=1}^{P}\sum_{n=1}^{N}y_{jn}$ is the grand total of all species abundances in **Y**.

As shown in Eq. (4), being a weighted average of single‐species values, β(**Y**) can be additively decomposed into the contribution of its constituting elements *w*_*j*_β(**y**_*j*_), such that the relative contribution of species *j* to overall β is $\sum_{n=1}^{N}w_{j}\times \beta \left(y_{j}\right)/\beta \left(Y\right)$.

## 4 Worked example

To illustrate how the proposed metric works, I used data from a belt transect across the beech timberline in central Italy. The data were collected by Di Giustino, Stanisci, Acosta, and Blasi (2002) on the west side of Majella, in the central Apennines, to investigate the vegetation dynamics at the timberline following grazing abandonment. The highest peak is Mt. Amaro (2,793 m) and the substrate consists mainly of carbonate rocks. Annual precipitation is about 1,500 mm, and mean annual temperature is 5–6°C with no dry season.

**Figure 1:**
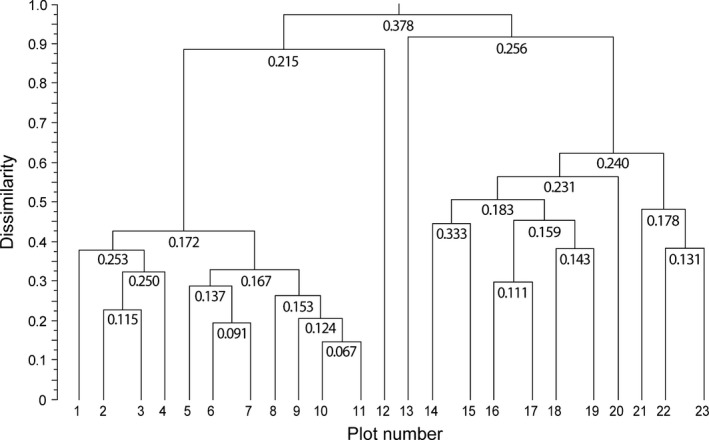
Dendrogram of the constrained cluster analysis of the belt transect used in the worked example. The clustering algorithm is based on the Chord distance calculated from the species abundance values in Table 1. For each node, the corresponding beta diversity value is shown

A belt transect composed of 23 quadrats of 1 m × 1 m was laid out across the upper forest line between the *Fagus sylvatica* forest and a dry *Brachypodium genuense* grassland at an altitude of 1,750 m on a gentle slope with deep soil (i.e., about 150 m below its potential upper limit; Stanisci, Lavieri, Acosta, & Blasi, 2001). In each quadrat, all vascular plants were recorded and the cover of each species was visually estimated by an experienced botanist using a 10% interval scale (Table 1). The quadrats were then hierarchically clustered using the Chord distance and a contiguity‐constrained segmentation method (see Legendre & Legendre, 2012). With this clustering method, only adjacent quadrats are considered for merging, such that the transect is divided into a hierarchical structure of compositionally homogeneous clusters of adjacent plots, or segments (Figure 1).

**Table 1:** Community composition matrix of the belt transect across the beech timberline in Central Italy used in the worked example

Species	Plot Number																							% β
	1	2	3	4	5	6	7	8	9	10	11	12	13	14	15	16	17	18	19	20	21	22	23	
*Fagus sylvatica*	6	6	6	6	6	7	7	7	6	6	6	2												20.066
*Polystichum aculeatum*	1				1																			2.033
*Epilobium montanum*	1					1	1																	2.543
*Lathyrus vernus*	1	1				1	1																	2.911
*Galium odoratum*	1			1						1	1													2.911
*Sedum album*		1																						1.305
*Micelis muralis*		1	1	1			1	1																3.175
*Sanicula europaea*				1																				1.305
*Hepatica nobilis*				1	1	1	1	1			1													3.355
*Ajuga reptans*				1								1	1											2.543
*Cephalanthera damasonium*				1																				1.305
*Cardamine bulbifera*					1		1																	2.033
*Ranunculus nemorosus*								1				1	1	1		1	1							3.355
*Campanula scheuchzeri*								1	1			1												2.543
*Poa nemoralis*												1												1.305
*Brachypodium genuense*												1	1	4	4	4	4	3	3	5	5	3	5	12.865
*Galium lucidum*												1	1		1				1					2.911
*Rumex acetosa*												1			1	1	1	1	1		1			3.465
*Hieracium sylvaticum*															1			1						2.033
*Cerastium arvense*													1			1	1	1	1	1	1	1	1	3.514
*Lotus corniculatus*																1	1	1	1	1	1	1	1	3.515
*Stachys timphaea*																	1							1.305
*Euphorbia cyparissias*																	1			1				2.033
*Armeria majellensis*																				1				1.305
*Potentilla rigoana*																				1				1.305
*Hieracium pilosella*																				1				1.305
*Festuca curvula*																				1		1	1	2.543
*Helianthemum nummularium*																					1	1		2.033
*Verbascum thapsus*																					1			1.305
*Avenula praetutiana*																					1		1	2.033
*Laserpitium garganicum*																					1			1.305
*Trifolium pratense*																					1	1	1	2.543

Columns are adjacent plots, and rows are species. The species abundances are a visual estimate of the species cover on a 10% interval scale. The last column shows the percent contribution of single species to overall beta diversity of the whole transect.

Finally, using Eq. (4), I calculated the beta diversity for each node of the dendrogram in Figure 1. For the calculation of the beta diversity of a given node, all species were weighted proportionally to their total cover within the corresponding segment. All calculations were performed with the R script available in Appendix S1.

## 5 Results

In the study area, like in many other regions of high grazing pressure in the central Apennines, the beech forest reaches the timberline giving rise to an abrupt contact with grasslands without the presence of an intermediate transition belt of shrub species, such as *Juniperus alpina, Arctostaphylos uva‐ursi, Rhamnus alpina, Rosa pendulina, Rubus idaeus* or *Lonicera alpigena* (Stanisci et al., 2001). Such abrupt contacts are usually found about 100–200 m below the potential upper limit of the treeline, in physiographic conditions which favor intense grazing activity. In such conditions, vegetation dynamics is blocked by disturbance and beech forest may spread only slowly to higher altitudes (Di Giustino et al., 2002).

As a result, the transect in Table 1 can be clearly divided into two main compositionally distinct clusters with only two transitional quadrats represented by plots 12 and 13 (Figure 1). As expected, the floristic homogeneity within each group of adjacent plots is generally high (i.e., beta diversity is low) and tends to decrease more or less gradually along the nodes of the dendrogram, meaning that community composition tends to become more and more “beta diverse” along the hierarchy of the dendrogram when the different groups of adjacent plots are merged into a higher‐level cluster. The highest compositional heterogeneity is associated with the upper node of the dendrogram when the forest plots are pooled with the grassland plots.

Looking at the contribution of single species to overall beta diversity (Table 1), we have that the dominant species *Fagus sylvatica* and *Brachypodium genuense* account for roughly one‐third (32.93%) of the beta diversity of the whole transect (i.e., to the beta diversity associated with the upper node of the dendrogram in Figure 1). By contrast, due to their low abundance, the 10 singleton species with just one presence in the whole transect (i.e., with β(**y**_*j*_) = 1) account for a mere 13.05% of total beta. However, weighting all species equally, the contribution of the singleton species raises to 44.63%, whereas the contribution of *Fagus sylvatica* and *Brachypodium genuense* decreases to 2.02% (data not shown). This emphasizes the crucial role of the weighting criteria for the calculation of a biologically reasonable beta diversity figure that conforms to the specific users’ requirements.

Rare species usually constitute an heterogeneous pool of occasional plants of low persistence and low fidelity of association with specific communities (Grime, 1998). As such, they are also quite unevenly distributed among the plots. Therefore, according to this general direct relationship between rarity and spatial unevenness, weighting the species by their abundances emphasizes the role of dominant species, reducing at the same time the relevance of occasional species with very low abundances. On the other hand, using equal weights for all species emphasizes the role of rare species irrespective of their overall abundances and their fidelity of association with specific community types.

## 6 Discussion

In this study, I introduced a method for calculating the beta diversity of a community composition table, which preserves the relative dispersion of abundances within species vectors. The proposed method allows to shed new light on the relationships between α, β, and γ diversity: α and γ are computed from single plot vectors **x**_*n*_ and from the vector of species sums **x**_+_ = [*y*_*j*+_], respectively, whereas β is computed from the species vectors **y**_*j*_. Hence, in a sense, α and γ are the warp, and β is the weft of the community composition table. The major difference between alpha, gamma, and beta diversity is that, for a fixed number of species, α and γ increase with increasing evenness, whereas for a fixed number of plots, β increases with decreasing evenness.

Being based on a weighted average of inverse evenness measures, β(**Y**) is very flexible and allows for various types of weighting methods, which can be determined depending on the specific ecological question. For presence/absence scores, a reasonable strategy may consist in weighting all species equally, whereas for abundance data, the species may be weighted proportionally to the row sums *y*_*j*+_.

From an ecological viewpoint, this weighting method is directly related to the mass‐ratio hypothesis of Grime (1998), which states that ecosystem processes, like water balance or nutrient cycling, are largely determined by the functioning of the dominant species and are relatively insensitive to the presence of less abundant species. This effect is dictated by the fact that, especially for autothrophs such as plants, a larger body mass involves major contribution to syntheses, resource fluxes, and degradative processes (Grime, 1998). Accordingly, if our aim consists in relating the amount of variation of the species composition in **Y** to the spatial organization of ecosystem functioning, weighting the species according to their abundances in the data table may represent an adequate choice. Alternatively, within a more functional context, the species weights *w*_*j*_ may also be set proportional to the average or minimum functional dissimilarity of *j* from the other species in the community composition table, such that more weight is given to the most functionally distinct species.

As highlighted by Anne Chao (pers. comm.), when the species weights are proportional to their abundances (i.e., *w*_*j*_ = *y*_*j*+_/*y*_++_) and the beta diversity of single‐species vectors β(**y**_*j*_) is calculated with Pielou's evenness, overall beta β(**Y**) is the same as the mutual information measure of beta diversity derived in Chao and Chiu (2016, Eq. 11c). This index, which is part of a larger parametric family of information‐theoretical measures of beta diversity, bridges the gap between the normalized variance of a community composition matrix (after removing the constraints by alpha, gamma, and total abundance) and traditional diversity decomposition methods (based on partitioning gamma diversity into alpha and beta components). Hence, the observed relationship between β(**Y**) and Chao and Chiu's beta highlights once again the connection between diversity theory and information‐theoretical measures.

Concerning the choice of an appropriate measure of evenness for calculating beta, in this study, I used the classical Pielou's evenness (see Jost, 2010). However, in ecology, there is a plethora of available evenness measures such that, according to Kvålseth (2015): “a researcher seeking an evenness index to use in a particular study is faced with a bewildering choice”. Extensive reviews of evenness measures and their properties can be found in Smith and Wilson (1996), Ricotta (2003), Tuomisto (2012), and Kvålseth (2015). While a variety of properties have been advocated for evenness, there does not appear to be any general consensus as to which is really necessary. With a focus on the measurement of beta diversity of single‐species vectors, an intuitively relevant property is the so‐called principle of transfers, which was introduced in econometrics by Dalton (1920) in the framework of income distribution. In its very essence, given a relative abundance distribution $\left(p_{1},p_{2},\ldots ,p_{Q}\right)$ and two objects *i* and *j* with relative abundances *p*_*i*_ > *p*_*j*_, evenness is increased if the quantity Δ is transferred from *p*_*i*_ to *p*_*j*_ so long as the transfer does not reverse the ranking of the two abundances *p*_*i*_ − Δ > *p*_*j*_ + Δ. Hence, consistently with our intuitive notion of beta diversity, the transfer property states that, for a given species, evenness is increased (beta is decreased) when the species abundance is transferred from one plot to another plot in which the species is less abundant. For mathematical details, see Patil and Taillie (1982) and Kvålseth (2015).

Another desirable property of β(**Y**) is its ability to be additively decomposed into species‐level contributions, thus enabling to highlight the relevance of single species to overall beta diversity. This property arises directly from the definition of β(**Y**) as the weighted average of the single‐species values β(**y**_j_). Therefore, it is preserved even if β(**Y**) is calculated with an evenness index other than Pielou's evenness. To the contrary, decomposing beta into plot‐level contributions is much less obvious, such that the role of specific plots in shaping overall beta diversity is best summarized by other methods; for example, by calculating the mean dissimilarity of a given focal plot from all other plots in **Y**. For deeper discussion on the decomposition of β(**Y**) into single‐plot contributions, see Appendix S2. Note that, for a single pair of plots, if β(**Y**) is calculated from presence/absence scores with equal weights *w*_*j*_ = 1/*P*, beta reduces to the well‐known Jaccard dissimilarity coefficient, whereas if the weights *w*_*j*_ are set proportional to the number of species presences in both plots, beta reduces to the Sørensen dissimilarity (proof in Appendix S3). As a result, β(**Y**) can be considered a multiple‐site dissimilarity measure *sensu* Diserud and Ødegaard (2007), thus bridging the gap between beta diversity, evenness, and dissimilarity. At the same time, the connection between evenness and dissimilarity gives rise to a new family of plot‐to‐plot (dis)similarity coefficients based on the rich arsenal of available evenness and concentration measures. In addition to species presence/absence scores, such evenness‐based dissimilarity measures can also include the species relative abundances and between‐species functional and phylogenetic resemblances (see Ricotta & Pavoine, 2015).

Can the proposed method be further generalized to include other approaches to the measurement of beta diversity? For example, can the method be extended to other multiple‐site dissimilarity coefficients, or can Pielou's evenness be generalized to include the entire family of information‐theoretical measures of beta diversity proposed by Chao and Chiu (2016)? These are critical questions, and their answers may provide valuable insights into the effects of ecological, evolutionary, and human‐driven mechanisms on community composition.

## Conflict of interest

None declared.
