# Supplementary material for: Of beta diversity, variance, evenness, and dissimilarity
Source: Ecol Evol. 2017 May 26;7(13):4835–43. doi: 10.1002/ece3.2980 (PMC5496569; doi:10.1002/ece3.2980)
Supplement: Supplementary file 1 [file ECE3-7-4835-s001.docx]

**Appendix 1:** Function *betaeve* for the calculation of the beta-diversity index introduced in the main text.

This program is free software: you can redistribute it and/or modify it under the terms of the GNU General Public License <http://www.gnu.org/licenses/>.

**Disclaimer:** users of this code are cautioned that, while due care has been taken and it is believed accurate, it has not been rigorously tested and its use and results are solely the responsibilities of the user.

**Description:** given a community composition matrix **Y** composed of *P* species (rows) × *N* plots (columns), the R function *betaeve* calculates the total beta-diversity of **Y** by averaging the concentration values (i.e. the complement of Pielou’s evenness values) of the single species vectors in **Y**.

**Dependencies:** the function requires the *vegan* package (Oksanen et al. 2016).

**Usage**: betaeve<-function(comm)

**Arguments**

*comm*: a *data.frame* of *P* species (rows) × *N* plots (columns) containing the abundances of all *P* species in the *N* plots.

**Output**

the function returns a *list* object composed of three elements:

1) ‘Average Beta’ = the overall beta-diversity of the community composition matrix **Y** calculated as the unweighted average of the single-species values of beta. For additional details refer to the main text.

2) ‘Species weights’ = a vector containing the single-species weights used for calculating the weighted beta-diversity of the community composition matrix **Y**. All weights are proportional to the total species abundances in **Y**.

3) ‘Weighted Beta’ = the overall beta-diversity of the community composition matrix **Y** calculated as the weighted average of the single-species values of beta.

**References**

Oksanen, J., Blanchet, F.G., Kindt, R., Legendre, P., Minchin, P.R., O'Hara, R.B., Simpson, G.L., Solymos, P., Stevens, M.H.H., Wagner, H. (2016) vegan: Community Ecology Package. R package version 2.3-3. https://CRAN.R-project.org/package=vegan.

**Function Syntax**

betaeve<-function(dataset){

require(vegan)

n_col<-ncol(dataset)

n_row<-nrow(dataset)

total <- apply(dataset, 1, sum)

species_weights<-total/sum(dataset)

rel_abu<- sweep(dataset, 1, total, "/")

h_shannon<-diversity(rel_abu, index = "shannon", MARGIN = 1)

pielou<-h_shannon/log(n_col)

pielou_com<-1-sum(pielou/n_row)

weighted_pielou<-1-sum(pielou*species_weights)

output<-list("Average Beta"=pielou_com,"Species weights"=species_weights, "Weighted Beta" = weighted_pielou)

print(output)

}

**Example**

data(dune) #Vegetation Dutch Dune Meadows freely available in the *vegan* package. Community composition matrix composed of 20 plots (matrix rows) and 30 species (matrix columns).

t_dune<-t(dune) #transpose the community composition matrix

betaeve(t_dune) #run the *betaeve* function

**Results**

$`Average Beta`

[1] 0.4811316

$`Species weights`

Achimill Agrostol Airaprae Alopgeni Anthodor Bellpere Bromhord Chenalbu

0.023357664 0.070072993 0.007299270 0.052554745 0.030656934 0.018978102 0.021897810 0.001459854

Cirsarve Comapalu Eleopalu Elymrepe Empenigr Hyporadi Juncarti Juncbufo

0.002919708 0.005839416 0.036496350 0.037956204 0.002919708 0.013138686 0.026277372 0.018978102

Lolipere Planlanc Poaprat Poatriv Ranuflam Rumeacet Sagiproc Salirepe

0.084671533 0.037956204 0.070072993 0.091970803 0.020437956 0.026277372 0.029197080 0.016058394

Scorautu Trifprat Trifrepe Vicilath Bracruta Callcusp

0.078832117 0.013138686 0.068613139 0.005839416 0.071532847 0.014598540

$`Weighted Beta`

[1] 0.2990298
