# Supplementary material for: Of beta diversity, variance, evenness, and dissimilarity
Source: Ecol Evol. 2017 May 26;7(13):4835–43. doi: 10.1002/ece3.2980 (PMC5496569; doi:10.1002/ece3.2980)
Supplement: Supplementary file 2 [file ECE3-7-4835-s002.docx]

**Appendix 2:** About the additive decomposition of *β*(**Y**) into single-plot contributions.

Given a community composition matrix $\mathbf{Y}=[y_{jn}]$ containing the presence/absence or the abundance values of *P* species (row vectors **y***_j_* = **y**_1_, **y**_2_, ... **y***_P_* of **Y**) in *N* plots (column vectors **x***_n_* = **x**_1_, **x**_2_ ... **x***_N_* of **Y**), let EVE be any evenness measure in the range 0–1. According to the main text, the beta-diversity of matrix **Y** is obtained as follows: first, the raw abundances $y_{jn}$ are normalized into relative values per row by dividing each value by the row sum $y_{j+}=\sum_{n=1}^{N} y_{jn}$ such that $p_{jn}=y_{jn}/y_{j+}$. Next, the beta-diversity of single species vectors is calculated from the normalized values *p_jn_* as:

$\beta(\mathbf{y}_{j})=1-\mathrm{EVE}(\mathbf{y}_{j})$ (1)

where $\mathrm{EVE}(\mathbf{y}_{j})$ is the evenness of row (species) *j*. For a fixed number of plots *N*, *β*(**y**_j_) = 1 if species *j* is present only in one plot with relative abundance equal to one, while *β*(**y**_j_) = 0 if *j* is present in all plots with relative abundance $1/N$. Finally, the beta-diversity of **Y** is defined as the weighted average of the single-species beta:

$\beta(\mathbf{Y})=\sum_{j=1}^{P} w_{j}\times\beta(\mathbf{y}_{j})=1-\sum_{j=1}^{P} w_{j}\times\mathrm{EVE}(\mathbf{y}_{j})$ (2)

with weights $0\leq w_{j}\leq1$ and $\sum_{j=1}^{P} w_{j}=1$.

The ability to additively decompose overall beta into species-level contributions arises directly from the definition of *β*(**Y**) as the weighted average of the single-species values *β*(**y**_j_). Therefore, this property is preserved independently of the evenness index used for calculating *β*(**y**_j_). To the contrary, a necessary requirement for decomposing overall beta into per-plot contributions is related to the property of the single-species values *β*(**y**_j_) to be additively decomposed into their constituting elements, such that $\beta\left(\mathbf{y}_{j}\right)=\sum_{n=1}^{N} \beta\left(y_{jn}\right)$, also known as sum-property. Therefore, the ability of *β*(**Y**) to be additively decomposed into plot-level contributions is index-dependent and hence less general than the ability to be decomposed into species-level contributions.

Given a measure of beta that conforms to the sum property, the beta-diversity of a single plot *β*(**x***_n_*) is then obtained as:

$\beta\left(\mathbf{x}_{n}\right)=\sum_{j=1}^{P} w_{j}\times\beta\left(y_{jn}\right)$ (3)

such that $\beta\left(\mathbf{Y}\right)=\sum_{n=1}^{N} \beta\left(\mathbf{x}_{n}\right)$. Eq. (3) thus implies that, like for the plot-level decomposition of SS(**Y**), the quantities *β*(*y_jn_*), which are derived at the species level, are then reassembled at the plot level. Whether this operation makes biological sense is left to the judgment of the practitioner.

Unfortunately, to the best of my knowledge, none of the evenness measures which conform to the principle of transfers (see Kvålseth 2015) is formulated such that their complement $\beta(\mathbf{y}_{j})=1-\mathrm{EVE}(\mathbf{y}_{j})$ can be additively decomposed into its (non-negative) constituting elements. For example, for single-species vectors, the complement of Pielou’s evenness $1-EVE\left(\mathbf{y}_{j}\right)=1-H\left(\mathbf{y}_{j}\right)/\log N$ can be additively decomposed into the contributions of single row elements *y_jn_* as:

$\beta\left(\mathbf{y}_{j}\right)=\frac{\log N-H}{\log N}=\sum_{n=1}^{N} \frac{p_{jn}\log\left(N\times p_{jn}\right)}{\log N}$ (4)

where $\beta\left(y_{jn}\right)=p_{jn}\log\left(N\times p_{jn}\right)/\log N$is the contribution of element *y_jn_* to *β*(**y**_j_), and $H\left(\mathbf{y}_{j}\right)=-\sum_{n=1}^{N} p_{jn}\log p_{jn}$ is the Shannon entropy.

However, for a given number of plots *N*, the term $\log\left(N\times p_{jn}\right)$ in Eq. (4) assumes non-negative values only for $p_{jn}\geq1/N$ with $p_{jn}=1/N$denoting perfect equitability. For relative abundances$p_{jn}<1/N$we have $\log\left(N\times p_{jn}\right)<0$ and hence $\beta\left(y_{jn}\right)<0$. In other words, while *β*(**y**_j_) is always non-negative in the range 0–1, the contribution of the single elements *y_jn_* to beta can be either positive or negative. Elements with relative abundances $p_{jn}$ larger than $1/N$ increase unevenness or beta-diversity; elements with relative abundances lower than $1/N$produce negative values of *β*(*y_jn_*), thus reducing beta-diversity. Therefore, those who believe the additive decomposition of beta into its constituting elements *β*(*y_jn_*) cannot produce negative values will find this approach unsatisfactory.

Likewise, as highlighted by Anne Chao (pers. comm.), when the species weights are proportional to their abundances (i.e. $w_{j}=y_{j+}/y_{++}$) and the beta diversity of single species vectors $\beta(\mathbf{y}_{j})$ is calculated with Pielou’s evenness, overall beta *β*(**Y**) is the same as the mutual information measure of beta diversity derived in Chao and Chiu (2016, Eq. 11c). This measure can be expressed as:

$\beta\left(Y\right)=\frac{1}{y_{++}\log N}\sum_{j=1}^{P} \sum_{n=1}^{N} y_{jn}\log\frac{y_{jn}}{\bar{y}_{j+}}$ (5)

where $y_{++}=\sum_{j=1}^{P} \sum_{n=1}^{N} y_{jn}$ is the grand total of all species abundances in **Y** and $\bar{y}_{j+}=\sum_{n=1}^{N} y_{jn}/N$.

According to Eq. (5), *β*(**Y**) can be decomposed into per-species and per-plot contributions such that the contribution from species *j* is $\beta\left(y_{j}\right)=\frac{1}{y_{++}\log N}=\sum_{n=1}^{N} y_{jn}\log\frac{y_{jn}}{\bar{y}_{j+}}$. Therefore, we have that overall beta is the sum of the contributions of *P* species $\beta\left(Y\right)=\sum_{j=1}^{P} \beta\left(y_{j}\right)$. At the same time, the contribution from plot *n* is $\beta\left(x_{n}\right)=\frac{1}{y_{++}\log N}=\sum_{j=1}^{P} y_{jn}\log\frac{y_{jn}}{\bar{y}_{j+}}$. In this case, overall beta is the sum of the contributions of *N* plots $\beta\left(Y\right)=\sum_{n=1}^{N} \beta\left(x_{n}\right)$.

However, in both cases, the term $\log\left(y_{jn}/\bar{y}_{j+}\right)$ in Eq. (5) is non-negative only for $y_{jn}/\bar{y}_{j+}\geq1$, meaning that element *y_jn_* can assume negative values. Therefore, it is easily shown that the plot-level contributions $\beta\left(x_{n}\right)$ can also assume negative values. While this does not prevent to calculate a relative plot-level value of beta $\beta\left(x_{n}\right)/\beta\left(Y\right)$, its biological meaning may not be accepted unanimously by ecologists.

Another line of attack may consist in substituting the notion of beta-diversity with its complement, beta-evenness or beta-equitability, which measures the degree of *similarity* in species composition among sampling units. According to this definition, the beta-equitability of the community composition matrix **Y** can be expressed as:

$EVE(\mathbf{Y})=\sum_{P} w_{j}\times EVE(\mathbf{y}_{j})$ (6)

Here, using Pielou’s evenness $EVE\left(\mathbf{y}_{j}\right)=H\left(\mathbf{y}_{j}\right)/\log N$, the beta-equitability of single rows can be additively decomposed as:

$EVE\left(\mathbf{y}_{j}\right)=\sum_{n=1}^{N} EVE\left(y_{jn}\right)=\sum_{n=1}^{N} \frac{-p_{jn}\log p_{jn}}{\log N}$ (7)

where all constituting elements EVE(*y_jn_*) assume non-negative values. Accordingly, the beta-equitability of a single plot EVE(**x***_n_*) can be then obtained as $EVE\left(\mathbf{x}_{n}\right)=\sum_{j=1}^{P} w_{j}\times EVE\left(y_{jn}\right)$ such that $EVE\left(\mathbf{Y}\right)=\sum_{n=1}^{N} EVE\left(\mathbf{x}_{n}\right)$.

The same approach can be used for additively partitioning a number of other evenness measures, such as the Simpson evenness $EVE\left(\mathbf{y}_{j}\right)=\sum_{n=1}^{N} p_{jn}\left(1-p_{jn}\right)/\left(1-1/N\right)=\sum_{n=1}^{N} p_{ij}^{2}/\left(1-1/N\right)$ (see Smith and Wilson 1996), or the index of Solomon (1979) $EVE\left(\mathbf{y}_{j}\right)=2\sum_{n=1}^{N} \left(\rho-1\right)p_{jn}^{*}/\left(N-1\right)$, where $p_{jn}^{*}$ are the relative abundances of species *j* in the *N* plots ranked in descending order such that $p_{j1}^{*}\geq p_{j2}^{*}\geq...\geq p_{jN}^{*}$ and  *ρ* are the corresponding ranks 1, 2, ..., *N*.

**References**

Chao, A., Chiu, C.H. (2016) Bridging the variance and diversity decomposition approaches to beta diversity via similarity and differentiation measures. Methods in Ecology and Evolution 7: 919–928.

Kvålseth, T.O. (2015) Evenness indices once again: critical analysis of properties. SpringerPlus 4: 232. DOI 10.1186/s40064-015-0944-4.

Smith, B., Wilson, J.B. (1996) A consumer’s guide to evenness indices. Oikos 76: 70–82.

Solomon, D.L. (1979) A comparative approach to species diversity. In: Grassle, J.F., Patil, G.P., Smith, W., Taillie, C. (Eds.) Ecological diversity in theory and practice. International Co-operative Publishing House, Fairland, Maryland, pp. 29–35.
