# Supplementary material for: Of beta diversity, variance, evenness, and dissimilarity
Source: Ecol Evol. 2017 May 26;7(13):4835–43. doi: 10.1002/ece3.2980 (PMC5496569; doi:10.1002/ece3.2980)
Supplement: Supplementary file 3 [file ECE3-7-4835-s003.docx]

**Appendix 3:** Proof that, for species presence/absence scores, if all weights *w_j_* are set equal to $1/P$, the overall beta *β*(**Y**) of a single pair of plots reduces to the Jaccard dissimilarity. Conversely, if the weights *w_j_* are set proportional to the number of species presences in both plots, *β*(**Y**) reduces to the Sørensen dissimilarity.

Given two plots *U* and *V* with the presence and absence data of *P* species, the Jaccard (1900) and the Sørensen (1948) dissimilarity coefficient are defined as:

$$Jac=\frac{b+c}{a+b+c}$$

and

$$Sør=\frac{b+c}{2a+b+c}$$

where *a*, *b* and *c* represent the matching/mismatching components of the well-known $2\times2$ contingency table: *a* is the number of species present in both plots, *b* is the number of species present only in plot *U*, and *c* is the number of species present only in plot *V* such that $a+b+c$ is the total number of species *P* in both plots.

Let EVE be any evenness index (not only the Pielou evenness), which takes on values between 0 and 1 and $\mathrm{EVE}(\mathbf{y}_{j})$the evenness of species vector $\mathbf{y}_{j}$. For a single pair of plots, $\mathrm{EVE}(\mathbf{y}_{j})$ takes the value one (denoting maximum evenness) if species *j* is present in both plots and the value zero (denoting minimum evenness) if *j* is present only in one plot, such that $\sum_{j\in U,j\in V} \mathrm{EVE}(\mathbf{y}_{j})=a$, $\sum_{j\in U,j\notin V} \mathrm{EVE}(\mathbf{y}_{j})=0$ and $\sum_{j\notin U,j\in V} \mathrm{EVE}(\mathbf{y}_{j})=0$.

Since $\beta(\mathbf{Y})=1-\sum_{j=1}^{P} w_{j}\times\mathrm{EVE}(\mathbf{y}_{j})$, using equal weights $w_{j}=1/P$ we have: $\beta(\mathbf{Y})=1-\sum_{j=1}^{P} 1/P\times\mathrm{EVE}(\mathbf{y}_{j})=1-a/\left(a+b+c\right)=\left(b+c\right)/\left(a+b+c\right)=Jac$.

On the other hand, setting the weights *w_j_* proportional to the number of species presences in both plots, we have that the weights associated to the species present in both plots are $w_{j}=2/\left(2a+b+c\right)$, while the weights associated to the species present in a single plot are $w_{j}=1/\left(2a+b+c\right)$, where $2a+b+c$is the number of species presences in both plots. Accordingly: $\beta(\mathbf{Y})=1-\frac{2}{2a+b+c}\sum_{j\in U,j\in V} \mathrm{EVE}(\mathbf{y}_{j})=1-2a/\left(2a+b+c\right)=\left(b+c\right)/\left(2a+b+c\right)=Sør$.

**References**

Jaccard, P. (1900) Contribution au problème de l’immigration post–glaciaire de la flore alpine. Bulletin de la Société Vaudoise des Sciences Naturelles 36: 87–130.

Sørensen, T. (1948) A method of establishing groups of equal amplitude in plant sociology based on similarity of species content and its application in analysis of the vegetation on Danish commons. Kongelige Danske Videnskabernes Selskabs Biologiske Skrifter 5: 1–34.
